# Dynamic Biomechanical Examination of the Lumbar Spine with Implanted Total Spinal Segment Replacement (TSSR) Utilizing a Pendulum Testing System

**DOI:** 10.1371/journal.pone.0057412

**Published:** 2013-02-25

**Authors:** Alan H. Daniels, David J. Paller, Sarath Koruprolu, Mark A. Palumbo, Joseph J. Crisco

**Affiliations:** Department of Orthopaedics, Warren Alpert Medical School of Brown University and Rhode Island Hospital, Providence, Rhode Island, United States of America; Georgia Health Sciences University, United States of America

## Abstract

**Background:**

Biomechanical investigations of spinal motion preserving implants help in the understanding of their *in vivo* behavior. In this study, we hypothesized that the lumbar spine with implanted total spinal segment replacement (TSSR) would exhibit decreased dynamic stiffness and more rapid energy absorption compared to native functional spinal units under simulated physiologic motion when tested with the pendulum system.

**Methods:**

Five unembalmed, frozen human lumbar functional spinal units were tested on the pendulum system with axial compressive loads of 181 N, 282 N, 385 N, and 488 N before and after Flexuspine total spinal segment replacement implantation. Testing in flexion, extension, and lateral bending began by rotating the pendulum to 5°; resulting in unconstrained oscillatory motion. The number of rotations to equilibrium was recorded and bending stiffness (N-m/°) was calculated and compared for each testing mode.

**Results:**

The total spinal segment replacement reached equilibrium with significantly fewer cycles to equilibrium compared to the intact functional spinal unit at all loads in flexion (p<0.011), and at loads of 385 N and 488 N in lateral bending (p<0.020). Mean bending stiffness in flexion, extension, and lateral bending increased with increasing load for both the intact functional spinal unit and total spinal segment replacement constructs (p<0.001), with no significant differences in stiffness between the intact functional spinal unit and total spinal segment replacement in any of the test modes (p>0.18).

**Conclusions:**

Lumbar functional spinal units with implanted total spinal segment replacement were found to have similar dynamic bending stiffness, but absorbed energy at a more rapid rate than intact functional spinal units during cyclic loading with an unconstrained pendulum system. Although the effects on clinical performance of motion preserving devices is not fully known, these results provide further insight into the biomechanical behavior of this device under approximated physiologic loading conditions.

## Introduction

**Figure 1:**
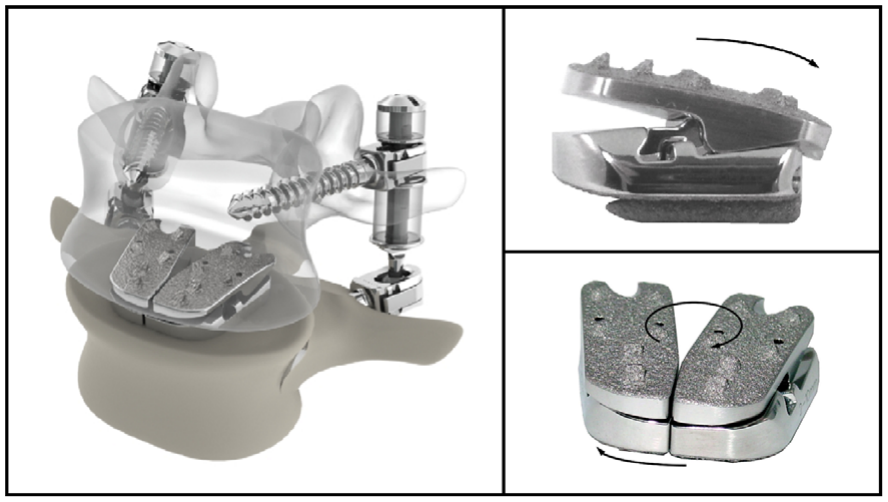
Total Spinal Segment Replacement device (Flexuspine^®^, Pittsburgh, PA). Composed of an interbody disc component with a metal-on-metal cobalt chromium articulation (*Core*) and posterior pedicle screw-based dynamic resistance component (*Dampener*).

There are numerous options for motion preservation surgery in the lumbar spine including nucleus pulposus replacement, total disc replacement (TDR), individual or bilateral facet replacement, flexible posterior rods, interspinous spacers, and total spinal segment replacement (TSSR) which replaces the disc in addition to the facet joints. The Flexuspine^®^ functional spinal unit (FSU) TSSR (Figure 1) was designed to provide an alternative to fusion by reestablishing mobility to an affected segment of the lumbar spine, and is implanted through a posterior only approach. It is a device composed of an interbody disc component with a metal-on-metal cobalt chromium articulation (*Core*) and posterior pedicle screw-based dynamic resistance component (*Dampener*)[1]. Flexuspine has been granted conditional approval by the FDA (as of April, 2010) to begin the initial U.S. phase of the Posterior Arthroplasty Safety (PASS) Study, but is not currently FDA approved for human use.

In addition to clinical studies, biomechanical investigations of motion preserving implants are necessary to complete our understanding of their *in vivo* behavior. The biomechanical properties of the ligamentous cadaver lumbar spine with and without implanted motion-preserving devices have been studied utilizing a wide variety of experimental protocols including constrained load-controlled testing, unconstrained load-controlled testing, unconstrained pure moment load-controlled testing, and displacement-controlled testing[2], [3], [4], [5], [6], [7], [8], [9], [10], [11], [12], [13], [14], [15], [16]. More recently, finite element analysis has been used to model spinal biomechanics both with and without motion preserving devices[2], [17], [18], [19], [20], [21]. However, many of the previously used protocols were limited in their ability to apply physiologic compressive loads greater than 200 N or to apply dynamic bending moments while allowing unconstrained three-dimensional motion.

To address these limitations, Crisco *et al* developed a novel pendulum testing system as a means to study the complex kinematics and the dynamic nature of the lumbar spine[22]. The pendulum apparatus is capable of dynamically applying physiologic compressive loads without constraining the motion of the FSU. The initial investigation utilizing the pendulum found that after an initial rotational perturbation, FSUs behaved as a dynamic, under-damped vibrating elastic system. Significant increases in bending stiffness and decreases in natural frequency were found with increasing compressive loading. Additionally, the number of cycles to equilibrium observed under pendulum testing is a marker of the energy absorbed by the FSU with fewer cycles to equilibrium indicating more rapid energy absorption. Previous investigation utilizing the pendulum system revealed that total disc replacement was less stiff, yet exhibited fewer cycles to equilibrium compared to the intact functional spinal unit during cyclic loading[23].

In this study, we hypothesized that the lumbar spine with implanted TSSR would exhibit decreased dynamic stiffness and more rapid energy absorption compared to native lumbar FSUs under simulated physiologic motion when tested with the pendulum system, similar to that exhibited by total disc replacement. We additionally aimed to determine the effects of various axial compressive loads on the dynamic biomechanical properties of native lumbar FSUs with implanted TSSR as compared to native lumbar FSUs.

## Methods

**Figure 2:**
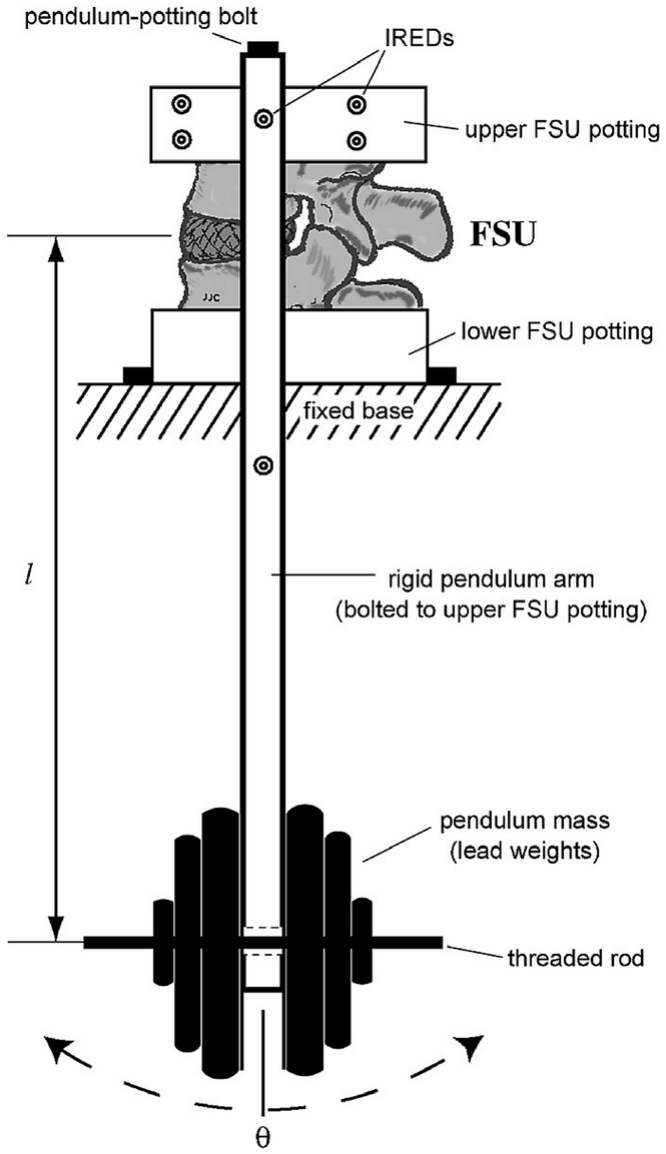
Pendulum testing apparatus [**22**].

Five de-identified unembalmed, frozen human lumbar FSUs were obtained from 4 cadavers (MedCure, Inc., Portland, OR), then thawed and tested individually (average age 71.3 years, range 59–91). Radiographic screening was performed to eliminate any samples with previous surgery, trauma, or pathologic lesion. One FSU from each level: L1/2, L2/3, L3/4, L4/5, L5/S1 was utilized for testing. Biomechanical testing of the FSUs was performed on a pendulum apparatus as described previously[22]. The pendulum system consists of the lower lumbar vertebra mounted on a rigid platform via its potting cup, and the pendulum arm (0.55 m) mounted to the upper vertebral body via its potting cup. The intervertebral disc or the TSSR serves as an unconstrained fulcrum with dead weights fixed to the lower end of the pendulum arm directly below the FSU (Figure 2).

Each intact FSU was tested on the pendulum system with axial compressive loads of 181 N, 282 N, 385 N, and 488 N, chosen to represent physiologic loading[24]. Testing began by manually rotating the pendulum to an initial angle of 5° and then releasing the pendulum, resulting in unconstrained oscillatory motion of the superior vertebra. Testing was performed in flexion, extension, right lateral bending, and left lateral bending. The three-dimensional motion of the superior vertebra relative to the inferior vertebra was measured at 200 Hz using an Optotrak 3020 (Northern Digital Inc., Ontario, Canada; RMS, accuracy to 0.1 mm and three-dimensional resolution to 0.01 mm). Six infrared-emitting diode markers were attached to the upper potting cup, and six to the lower potting cup. Custom NDI First Principles (Northern Digital Inc., Ontario, Canada) software was used to track the marker position of the upper vertebral body with respect to the lower vertebral body. Each test was repeated twice.

The motion data as the spine oscillated was collected until angular motion was <0.1°, at which point the cycles from initial perturbation to equilibrium were collected. The number of cycles to equilibrium of each specimen at each compressive load was averaged for flexion, extension, right and left lateral bending.

The mean dynamic bending stiffness (N-m/°) was calculated from the time series for each specimen at each compressive load. The dynamic bending stiffness of each specimen at each compressive load was averaged for flexion, extension, right and left lateral bending.

After initial testing of the intact spines, the FSUs underwent Flexuspine TSSR implantation. The FSUs were then re-tested on the pendulum apparatus with the same loading protocol.

In addition to pendulum testing, pure moment testing was performed on all specimens before and after TSSR implantation to determine quasi-static ROM and bending stiffnesses. A 400 N follower load was used during moment applications (0±6 Nm) at a test frequency of 0.1 Hz[24] with a biaxial servohydraulic load frame (model 8521S; Instron Corp., Canton, MA). Specimen holders and attachment plates were indexed in 45° increments about two perpendicular axes so that the FSU could be positioned at the various moment axes. A six-channel load cell (model MC3-6-1000; AMTI, Watertown, MA) acquired load and moment data about three orthogonal axes while rotary and linear motions about the vertical axis were measured with the load frame transducers[25]. Testing of FSUs involved applying positive and negative pure moments (0± 6Nm sinusoidal waveform with 0.1 Hz frequency) in right and left axial rotation, flexion, extension, and right and left lateral bending using a standard flexibility protocol to apply pure moments using test methods and fixtures that were published by Spenciner *et al* [25].

To compare statistical difference between the intact FSU and implanted TSSR samples for average cycles to equilibrium and dynamic stiffness, a two factor (treatment and compressive load) repeated measures ANOVA was performed (SigmaPlot 12.0, SYSTAT, San Jose, CA). In the event of statistical differences, a Tukey post hoc test was administered. For the pure moment Instron testing, the significance of the differences for each load by outcome variable between the intact spine and TSSR groups was calculated using a paired t-test. In all instances, statistical significance was set to p<0.05, *a priori*.

## Results

### Average number of cycles to equilibrium

**Figure 3:**
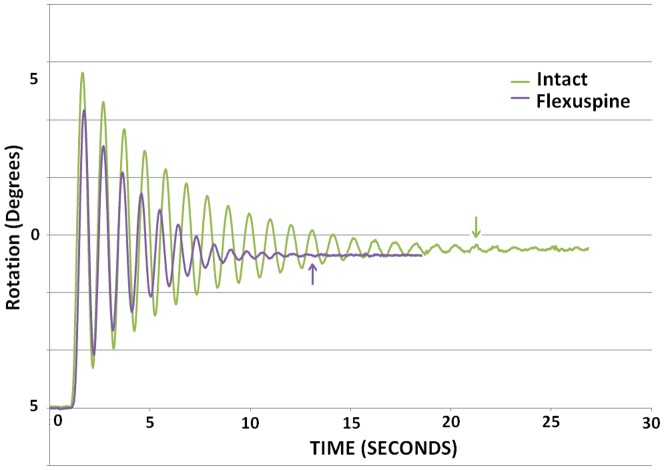
Typical rotation profile for an FSU before and after TSSR. Arrows indicate cycles to equilibrium.

The motion of the intact FSUs with and without implanted TSSR exhibited that of an under-damped, vibrating elastic system at each compressive load (Figure 3).

**Figure 4:**
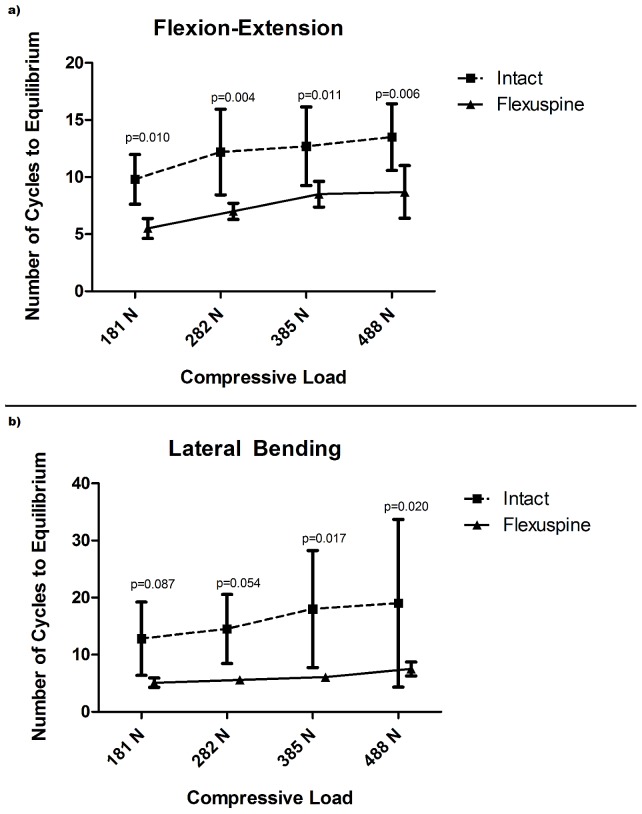
Pendulum testing results for mean cycles to equilibrium. Flexion (4a). Lateral bending (4b). Significantly fewer cycles to equilibrium for the TSSR indicate more rapid energy absorption for the TSSR compared to the intact FSU.

The average number of cycles to equilibrium increased with increasing compressive load for both flexion/extension testing, as well as lateral bending testing for the intact FSU and TSSR specimens. In flexion/extension testing with increasing load from 181 N to 488 N, the average number of cycles to equilibrium of the intact FSU specimens increased from 9.8 to 13.5. After TSSR implantation with increasing loads from 181 N to 488 N, the average number of cycles to equilibrium increased from 5.5 to 8.7. These results were significantly higher for the intact FSU versus TSSR specimens at all loads tested (p<0.011) (Figure 4a).

In lateral bending testing with increasing load from 181 N to 488 N, the average number of cycles to equilibrium of the intact FSU specimens increased from 12.8 to 19.0. After TSSR implantation with increasing load from 181 N to 488 N, the average number of cycles to equilibrium increased from 5.1 to 7.5. The TSSR exhibited significantly fewer cycles to equilibrium compared to the intact FSU at 385 N and 488 N (p = 0.017, p = 0.020, respectively), but not at 181 N or 282 N (p = 0.087, 0.054, respectively) (Figure 4b).

### Dynamic Bending Stiffness

**Figure 5:**
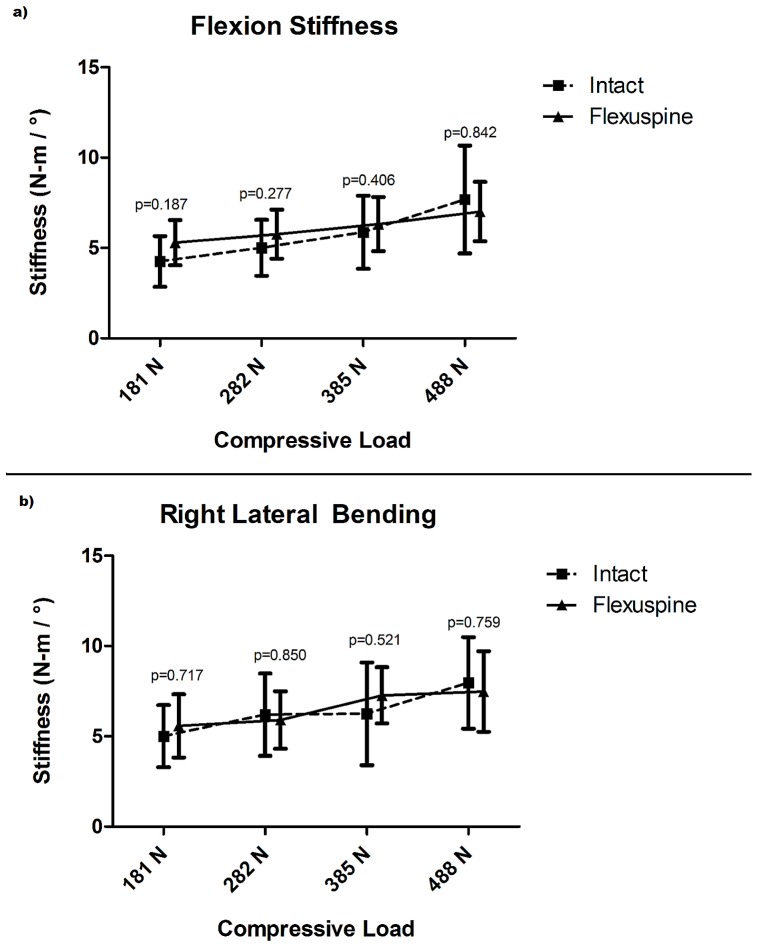
Pendulum testing results for mean bending stiffness (N-m/°). Flexion (5a). Lateral bending (5b). Results indicate similar stiffness profile for intact FSU and TSSR constructs.

The mean dynamic bending stiffness determined by pendulum testing in flexion, extension, left lateral bending, and right lateral bending significantly increased with increasing load for both the intact FSU and TSSR specimens (p<0.018). In flexion testing with increasing load from 181 N to 488 N, the mean bending stiffness of the intact FSU specimens increased from 4.1 N-m/° to 6.8 N-m/°. After TSSR implantation with increasing load from 181 N to 488 N, the mean bending stiffness increased from 5.3 N-m/° to 7.0 N-m/°, and was not significantly different between the intact FSU and TSSR constructs (p>0.186). (Figure 5a).

In extension testing, with increasing load from 181 N to 488 N, the mean bending stiffness of the intact FSU specimens increased from 4.0 N-m/° to 6.3 N-m/°. After TSSR implantation with increasing load from 181 N to 488 N, the mean bending stiffness increased from 5.3 N-m/° to 6.5 N-m/°, and was not significantly different between the intact FSU and TSSR constructs (p>0.112).

In right lateral bending testing with increasing load from 181 N to 488 N, the mean bending stiffness of the intact FSU specimens increased from 5.0 N-m/° to 8.0 N-m/°. After TSSR implantation with increasing load from 181 N to 488 N, the mean bending stiffness increased from 5.6 N-m/° to 7.5 N-m/°, and was not significantly different between the intact FSU and TSSR constructs (p>0.521) (Figure 5b).

In left lateral bending testing, with increasing load from 181 N to 488 N, the mean bending stiffness of the intact FSU specimens increased from 5.0 N-m/° to 7.4 N-m/°. After TSSR implantation, with increasing load from 181 N to 488 N, the mean bending stiffness increased from 5.5 N-m/° to 7.7 N-m/°, and was not significantly different between the intact FSU and TSSR constructs (p>0.698).

### Quasi-Static ROM and Bending Stiffness

The range of motion of the intact FSU in flexion/extension was 5.17°, compared to 5.82° for the TSSR (p = 0.478). The stiffness in flexion for the intact FSU was 2.5 N-m/° as compared to 2.4 N-m/° for the TSSR (p = 0.887). The stiffness in extension for the intact FSU was 3.9 N-m/° as compared to 2.9 N-m/° of the TSSR; this difference was not statistically significant (p = 0.152).

The range of motion of the intact FSU in lateral bending was 6.4°, compared to 5.8° for the TSSR (p = 0.813). The stiffness in right lateral bending for the intact FSU was 2.7 N-m/°, which was statistically similar to the TSSR stiffness of 3.3 N-m/° (p = 0.514). The stiffness in left lateral bending for the intact FSU was 2.5 N-m/°, which was also statistically similar to the TSSR stiffness of 3.3 N-m/° (p = 0.534).

## Discussion

This study examined the dynamic biomechanical properties of cadaver FSUs with and without implanted TSSR utilizing a pendulum testing apparatus and large compressive loads. Under pendulum testing with increasing axial loading, the bending stiffness and number of cycles to equilibrium increased for both the intact FSU and the TSSR. The number of cycles to equilibrium was significantly decreased following TSSR implantation at all loads in flexion/extension and loads of 385 N and above in lateral bending. This decrease in the number of cycle to equilibrium indicates more rapid energy absorption for the specimens with implanted TSSR as compared to intact FSUs.

The energy absorption characteristics of motion preserving implants have potential implications in the study of implant wear and particle formation, implant-bone interface reaction, and adjacent segment degeneration. This study did not evaluate where in the FSU that the energy absorption occurred. Energy absorption may occur at the bearing surface, the implant-bone interface, in the posterior silicone dampeners, or in the native intact anatomical structures, and is the subject of ongoing research for other spinal motion preserving devices[26], [27], [28].Under pendulum testing, increasing axial loading was significantly associated (p<0.018) with increasing stiffness in flexion, extension, and lateral bending for both intact and TSSR implanted FSUs. No statistically significant differences in bending stiffness were found between the intact FSU and the TSSR construct. In this study, we found an increase in stiffness for intact FSUs from 4.1 N-m/° to 6.8 N-m/° with an increase in loading from 181 N to 488 N. This range falls within the previously reported range of stiffness under compressive loading[5], [6], [12], [22], [23], [24]. Crisco *et al* [22] reported an increase in stiffness of 1.7 N-m/° to 3.5 N-m/° with loads ranging from 78 N to 488 N, while Miller *et al* [12] reported an increase in stiffness of 6 N-m/° to 11 N-m/° with bending loads ranging from 60 N to 95 N.

In a previous study, the biomechanical behavior of the ProDisc-L TDR was examined with the same pendulum apparatus and protocol as was conducted in this investigation[23]. In that study, the mean cycles to equilibrium in flexion/extension testing for the implanted TDR ranged from 7.1 to 11.5, as compared to 5.5 to 8.7 for the TSSR in this study. The TSSR thus may absorb more energy compared to the TDR under approximated physiologic loading conditions, although no statistical comparison of the results between the 2 studies was performed. In addition, the mean dynamic bending stiffness of the TDR in flexion ranged from 2.1 N-m/° to 3.6 N-m/°, as compared to 5.3 N-m/° to 7.0 N-m/° for the TSSR. Again, no statistical comparison was performed, but the TSSR may exhibit higher stiffness as compared to the TDR under pendulum testing. The posterior pedicle screw based dampeners may be the cause of the differences in biomechanical behavior between the TDR and TSSR, which may have implications for implant wear and adjacent level degeneration.

In addition to pendulum testing, we also performed pure moment testing in a quasi-static manner. Pure moment testing mimicked the pendulum results in some test modes, yet we did not perform a statistical comparison of the bending stiffness results due to differences in the range of motion tested and the methods by which we calculated stiffness. Although no direct comparison is valid, it is interesting to examine the data from both testing systems. Pendulum testing at 385 N in flexion revealed a bending stiffness of 6.3 N-m/° for the intact FSU and 5.6 N-m/° for the TSSR (p = 0.406), while pure moment testing at 400 N revealed a bending stiffness of 2.5 N-m/° for the intact FSU and 2.4 N-m/° for the TSSR (p = 0.877). Pendulum testing at 385 N in lateral bending revealed a bending stiffness of 6.3 N-m/° for the intact FSU and 7.3 N-m/° for the TSSR (p = 0.521), while pure moment testing at 400 N revealed a bending stiffness of 2.7 N-m/° for the intact FSU and 3.3 N-m/° for the TSSR (p = 0.514). The results of the pendulum testing system and the pure moment testing system differed in exact value for bending stiffness, although the trends were similar. The larger difference between the intact FSU and the TSSR in bending stiffness calculated from pendulum testing compared to pure moment testing suggests that the pendulum testing system may be able to detect small differences in stiffness not detected by pure moment testing.

In this study, we found that the bending stiffness of the TSSR was statistically similar to the intact FSU. The primary theoretical advantage of lumbar spine motion preserving implants over spinal fusion is to prevent adjacent segment disease; this can presumably be accomplished through replication of intact FSU stiffness and motion parameters. At this point, the effects of the bending stiffness of motion preserving devices on affected and adjacent segments are not completely understood. The long-term clinical effects of motion preserving spine surgery on adjacent levels are being investigated although long-term data is not yet available[29], [30], [31].

Numerous studies utilizing finite element analysis have examined the effects of motion preserving implants, fusions, and cementation techniques on stiffness at the treated and adjacent levels.[2], [11], [17], [18], [19], [20], [21], [32]. Rohlmann *et al* assessed ‘optimal’ stiffness of a pedicle screw-based motion preservation system[33]. They proposed that a spinal motion preserving implant system should optimally fulfill two tasks: allow physiological motion, and reduce load on adjacent spinal structures. They proposed an optimal axial stiffness of the longitudinal rods of 50 N/mm. To our knowledge, this type of finite element analysis has not been performed for a TSSR. The optimal stiffness of motion preserving implants is not truly known, and may need to be individualized for the patient undergoing this type of surgery.

The Flexuspine FSU TSSR device is not currently FDA approved, although conditional approval to begin human testing in the United States has been granted. In addition, prospective nonrandomized clinical data from 24 patients 12 months following implantation of the Flexuspine device has been reported with promising initial clinical results[1]. Interestingly, similar constructs have been implanted on an off-label manner with total disc arthroplasty combined with flexible posterior rod constructs with favorable clinical results reported[34] Further clinical and biomechanical research is clearly needed to assess the performance of spinal motion preserving devices such as TSSR.

This study had several possible limitations. We did not assess the coupled, three-dimensional motion of the FSUs after an initial perturbation, and only reported motion in the direction of the perturbation. However, the motion of intact FSUs tested on the pendulum has been shown to be dominated by the direction of the initial perturbation. An additional limitation was the lack of assessment of the level of disc degeneration of the intact FSUs. Significant degeneration of the disc and facet joints affects FSU stiffness[35], [36], [37], thus it is difficult to assess if the TSSR in this study mimicked healthy or degenerated FSUs. This lack of degeneration assessment may have led to the discrepancy between stiffness values between this investigation and the first pendulum investigation[22]. Furthermore, environmental factors such as body temperature and lubrication may affect *in vivo* TSSR performance, and this study did not examine any of these factors. Another possible limitation of this study includes the repeat testing performed on the pendulum and pure moment testing apparatuses, which may have damaged the specimens, and no post-testing assessment for specimen damage or implant loosening was performed.

Additionally, in this study we determined the rate of energy absorption based on the number of cycles to equilibrium. The number of cycles to equilibrium is related to the damping factor (which is a function of the damping coefficient) as well as the system inertia and the stiffness, both of which increase with increasing compressive load. Thus, a direct comparison of energy absorption is only possible under the same axial loading conditions and stiffness. In this investigation, stiffness of the intact FSU and TSSR constructs were found to be similar, thus our conclusions regarding the rate of energy absorption were based solely on number of cycles to equilibrium. If this were not the case, the relationship between energy dissipation rate and the number of cycles to equilibrium would be less obvious. Further biomechanical study may examine where energy absorption in intact FSUs and motion preserving devices is occurring. In addition, clinical studies with long term follow-up are essential to monitor rates of adjacent segment degeneration and implant related complications in patients with implanted TSSR devices.

This study examined the biomechanical performance of an implanted TSSR in the cadaver lumbar spine on a pendulum testing system. Our data provide additional insight into the ability of the pendulum testing apparatus to evaluate motion preserving spinal implants in simulated physiologic loading situations. Lumbar FSUs with implanted TSSR were found to have similar stiffness, but absorbed energy more rapidly than intact FSUs during cyclic loading with the unconstrained pendulum testing system. Studies such as this are important in the ongoing evaluation and development of spinal motion preserving implants.

## Supporting Information

Text S1 **Source of** **Figure 2** **** [**22**] **.**
(PDF)
