# Supplementary material for: Dynamic Biomechanical Examination of the Lumbar Spine with Implanted Total Spinal Segment Replacement (TSSR) Utilizing a Pendulum Testing System
Source: PLoS One. 2013 Feb 25;8(2):e57412. doi: 10.1371/journal.pone.0057412 (PMC3581457; doi:10.1371/journal.pone.0057412)
Supplement: Text S1 — Source of Figure 2 [22] . (PDF) [file pone.0057412.s001.pdf]

ELSEVIER

Journal of Biomechanics 40 (2007) 2767–2773

JOURNAL OF BIOMECHANICS

www.elsevier.com/locate/jbiomech
www.JBiomech.com

The dynamic flexion/extension properties of the lumbar spine *in vitro* using a novel pendulum system

Joseph J. Crisco*, Lindsey Fujita, David B. Spenciner

Bioengineering Laboratory, Department of Orthopaedics, Brown Medical School/Rhode Island Hospital, 1 Hoppin Street, Coro West, Suite 404, Providence, RI 02903, USA

Accepted 14 December 2006

Abstract

The biomechanical properties of the ligamentous cadaver spine have been previously examined using a variety of experimental testing protocols. Ongoing technical challenges in the biomechanical testing of the spine include the application of physiologic compressive loads and the application of dynamic bending moments while allowing unconstrained three-dimensional motion. The purpose of this study was to report the development of a novel pendulum apparatus that addressed these challenges and to determine the effects of various axial compressive loads on the dynamic biomechanical properties of the lumbar functional spinal unit (FSU). Lumbar FSUs were tested in flexion and extension under five axial compressive loads chosen to represent physiologic loading conditions. After an initial rotation, the FSUs behaved as a dynamic, underdamped vibrating elastic system. Bending stiffness and coefficient of damping increased significantly as the compressive pendulum load increased. The apparatus described herein is a relatively simple approach to determining the dynamic bending properties of the FSU, and potentially disc arthroplasty devices. It is capable of applying physiologic compressive loads at dynamic rates without constraining the kinematics of the joints, crucial requirements for testing FSUs *in vitro*.
© 2007 Elsevier Ltd. All rights reserved.

Keywords: Pendulum; Dynamic; Unconstrained; Biomechanics; Lumbar functional spinal unit

1. Introduction

During daily activities, the human spine functions dynamically under complex loading, including large compressive loads (Nachemson et al., 1986). Understanding how the spine responds to these loads is crucial to advances in the understanding of spinal mechanics, clinical care and surgical treatment. The majority of studies on spinal mechanics have focused on the *in vitro* biomechanical properties, either of spinal segments, functional spinal units (FSUs), or individual vertebrae, since studying the biomechanical properties of the spine *in vivo* is extremely difficult.

A variety of apparatuses and protocols have been employed to study the *in vitro* biomechanical properties of the spine (Edwards et al., 1987; Goel et al., 1988, 1995; Hirsch and Nachemson, 1954; Izambert et al., 2003; Janevic et al., 1991; Markolf and Steidel, 1970; Miller et al., 1986; Panjabi et al., 2000; Patwardhan et al., 1999; Schultz, 1979; Spenciner et al., 2003). Conventional protocols include displacement-controlled testing (Edwards et al., 1987; Goel et al., 1995), constrained, load-controlled testing (Goel et al., 1985, 1995), and unconstrained, load-controlled testing (Goel et al., 1988; Panjabi, 1977, 1988; Wilke et al., 1994, 2001). However, experimental design constraints in these protocols have limitations in determining the mechanical properties of the spine. Displacement-controlled testing can lead to unwanted specimen damage since failure loads are easily reached in certain directions, even with minimal displacement. More importantly, the motion of the spine is constrained to the degree of freedom of the displacing actuator. Load-controlled testing has the advantage of readily applying compressive loads. However, if the spine specimen undergoes large deformations, such as those associated with multiple-level testing, bending moments applied to the spine become a function of the ensuing

*Corresponding author. Tel.: +1 401 444 4231; fax: +1 401 444 4418.
E-mail address: Joseph_Crisco@brown.edu (J.J. Crisco).

0021-9290/$ - see front matter © 2007 Elsevier Ltd. All rights reserved.
doi:10.1016/j.jbiomech.2006.12.013

motion and the spine level. Several studies, therefore, have focused on the application of pure moments in an attempt to improve load-controlled testing. For the moment to remain pure, the apparatus must be designed such that the point of application does not restrict or constrain motion. Several systems have been developed to apply moments in an unconstrained fashion, however, these systems perform only quasi-static loading (Grassmann et al., 1998; Schultz, 1979; Wilke et al., 1994). A method for applying physiologic compressive preloads under dynamic loading conditions has been described, but is limited to extension and flexion motions (Patwardhan et al., 1999). Given the complex kinematics and the dynamic nature of the spine, an apparatus that is cost effective and capable of applying both physiologic compressive loads and a variety of dynamic bending moments without constraining the motion of the FSU would be a significant advance. A pendulum system has the potential to meet these goals.

A pendulum system with the interphalangeal joint as a fulcrum was first used to study synovial joint lubrication (Jones, 1936). Charnley (1959) used a pendulum to assist him in the design of his artificial hip joints, while Unsworth et al. (1975) used a pendulum with the ability to apply sudden loads to demonstrate the various modes of joint lubrication. Although the intervertebral joint is not a synovial joint, and though no work has recently been done with this methodology, the use of a pendulum system to examine the mechanical behavior of the spine is attractive. A pendulum allows for the application of physiologic compressive loads, the dynamic application of bending moments, and unconstrained motion thereby providing a realistic simulation of *in vivo* loading conditions. To our knowledge, no study has previously reported the dynamic *in vitro* biomechanical properties of FSUs utilizing various loads with unconstrained motion. The purpose of this study was to determine the dynamic, *in vitro* mechanical response of cadaver lumbar FSUs under various axial compressive loads, using a novel unconstrained three-dimensional pendulum system.

2. Methods

2.1. Specimen preparation

A total of five thoracolumbar FSUs (three from level T12/L1, one from level L2/L3, and one from level L4/L5) were obtained from four unembalmed human spines (average age 57.3±9.9 years). FSUs were prepared by removing all residual musculature but leaving the ligamentous structures intact. Both the superior and inferior vertebrae were cast in a urethane-molding compound within 89 mm diameter cylindrical aluminum potting cups. To provide secure fixation while maintaining full range of motion of the facets, the vertebral bodies were potted to the pars interarticularus. Specimens were kept frozen until the day of testing, at which point they were completely thawed. During testing, the FSUs were kept moist with saline-soaked gauze. For this study, the coordinate system comprised three orthogonal anatomic axes with the origin at the intersection of the mid-sagittal plane of the disc, the mid-transverse plane of the disc, and a frontal plane posterior to the anterior wall of the disc by $\frac{2}{3}$ of the disc depth. The axes were considered positive with respect to the specimens' left ($+X$), superior ($+Y$), and anterior ($+Z$) directions.

2.2. Pendulum apparatus

A novel pendulum apparatus was built with the intervertebral disc of the FSU serving as an unconstrained fulcrum (Fig. 1). The lower vertebral body was mounted via its potting cup to a rigid platform. The pendulum (steel square tubing, 3.81 cm × 3.81 cm) was mounted to the upper vertebral body via its potting cup. The pendulum was an open rectangular shape (66 cm long × 23 cm wide) oriented in the frontal plane. This open shape permitted the pendulum to be mounted to the FSU with its weights directly below the FSU.

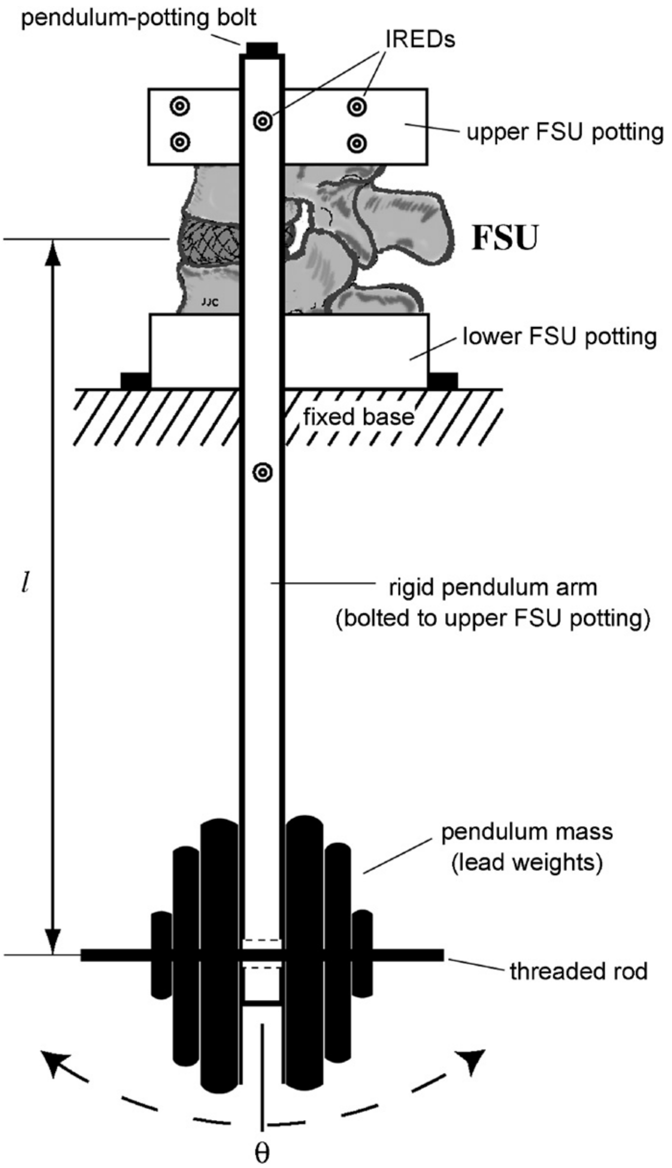

Fig. 1. Schematic of the pendulum apparatus showing the lower potted vertebra rigidly fixed and a pendulum arm fixed to the upper potted vertebra so that the intervertebral joint served as the fulcrum. The length of the pendulum (l) was fixed and the compressive load applied to the FSU was varied by changing the weight of the pendulum mass. The pendulum was set in motion by manually rotating it to 5° in extension and then releasing it. The three-dimensional motion of the pendulum (θ) was tracked using the infrared-emitting diode (ired) markers and the relative motion of the upper FSU with respect to the lower FSU calculated.

Axial compressive loads were applied to the FSUs by increasing the weight of the pendulum. Two aluminum plates spanned the lower portion of the pendulum arm, with a threaded steel rod at the center of each aluminum plate. Pairs of lead weights (mass 5.2 and 10.3 kg) were loaded on and removed from the steel rod symmetrically and carefully by hand to prevent any potentially damaging rotations. Each specimen was tested under five axial loads of 78, 181, 282, 385, and 488 N, achieved with various combinations of weights. The inertia magnitudes (I) about the intervertebral disc and the length of the pendulum from the intervertebral disc to the center of mass of the pendulum (l) were 0.98, 3.3, 5.7, 8.0, 10.6 kg m^2 and 0.35, 0.42, 0.44, 0.45, 0.46 m, respectively. These loads were chosen in an attempt to mimic those that the lumbar spine supports in a person weighing between 70 and 80 kg (Hirsch and Nachemson, 1954).

The three-dimensional motion of the superior vertebra relative to the inferior vertebra was measured at 30 Hz using an Optotrak 3020 three-dimensional motion tracking system (Northern Digital Inc., Ontario, Canada; RMS accuracy to 0.1 mm and three-dimensional resolution to 0.01 mm). Six infrared-emitting diode (ired) markers were attached to the upper potting cup and pendulum arm, and six to the lower FSU (Fig. 1). Custom NDI 6D Architect software (Northern Digital Inc., Ontario, Canada) was used to define the markers with respect to the coordinate system defined above in the inferior vertebra.

Testing began by manually rotating the superior vertebra to an initial angle of approximately 5° in extension using a thin, flexible cord attached to the lower portion of the pendulum. Releasing the cord resulted in the unconstrained oscillatory motion of the superior vertebra. Each test was repeated twice.

2.3. Data analysis

For each test, the average period (τ), natural frequency (ω), dynamic bending stiffness (k), and coefficient of damping (Q) were calculated and the values from both tests of each specimen at each compressive load were averaged. Since there were substantial regions of the time vs. rotation plots where the period remained relatively constant, the period could be determined graphically as the average of the first five cycles. The dynamic bending stiffness and coefficient of damping were calculated by modeling the rotation of the superior vertebra as a vibrating physical pendulum with a single degree of freedom (θ), linear damping (Q) and linear stiffness (k). The torque equilibrium equation about the fulcrum can be written

$$I\ddot{\theta} + Q\dot{\theta} + k\theta + mgl\sin\theta = 0, \quad (1)$$

where I is the moment of inertia of the pendulum, m is the total mass of the pendulum, l is the length of the pendulum from the center of the intervertebral disc to the center of mass of the pendulum, and g is the gravitational constant. Using the small angle approximation, and recasting as a vibrating system

$$\ddot{\theta} + \frac{Q}{I}\dot{\theta} + \frac{(k + mgl)}{I}\theta = 0 \quad (2)$$

and expressing the natural frequency (ω) as

$$\omega = \sqrt{\frac{k + mgl}{I}} \quad (3)$$

allows bending stiffness to be written explicitly:

$$k = \omega^2 I - mgl, \quad (4)$$

where the natural frequency (ω) was calculated as 2π times the inverse of the period. The damping coefficient (Q) was solved for by least-squares fitting the exponential decay function for an underdamped vibrating system to the peak rotation values using custom MATLAB software (Mathworks Inc. Natick, MA):

$$\theta = \theta_0 e^{-Qt/2I}, \quad (5)$$

where θ_0 is the initial rotation of the pendulum arm. The peak values for flexion and for extension were fit separately.

2.4. Verification study

In an attempt to verify that our approach to determine stiffness and damping values was valid and not influenced by secondary factors such as the mass of the pendulum, we replaced the FSU with a mechanical model whose intervertebral disc was simulated by rotary bearings. We assumed that this mechanical model would have negligible stiffness and negligible damping values. Tests were performed using methods identical to those described above.

2.5. Statistical analysis

A linear regression analysis was performed to determine the influence of compressive pendulum load on period, natural frequency, dynamic bending stiffness, and coefficients of damping by plotting the values for each variable ($n = 5$) at each compressive load values. In all cases, the level of statistical significance was set to 0.05 *a priori*.

3. Results

The motion of the FSU exhibited that of an underdamped vibrating elastic system at each compressive load (Fig. 2). The direction of the motion was dominated by flexion/extension rotations, which was the initial direction. Rotations in other directions occurred typically as the magnitude of flexion/extension decreased or they appeared and then damped out.

The period remained relatively constant through the approximately first 11 cycles and was found to increase linearly ($R^2 = 0.89$, $P = 0.01$) with increasing compressive load. The period of the FSU pendulum in flexion/extension increased from an average of 0.61 to 1.00 s as the compressive load increased from 78 to 488 N (Table 1). Accordingly, the natural frequency of the pendulum as it rotated the FSU in flexion/extension decreased from an average of 10.4 to 6.3 s^{-1} as the compressive load increased from 78 to 488 N.

Increasing the compressive pendulum load correlated significantly with a linear ($R^2 = 0.98$, $P < 0.01$) increase in the bending stiffness of the FSUs (Fig. 3). The bending stiffness at the lowest load averaged 1.7 Nm/° and at the

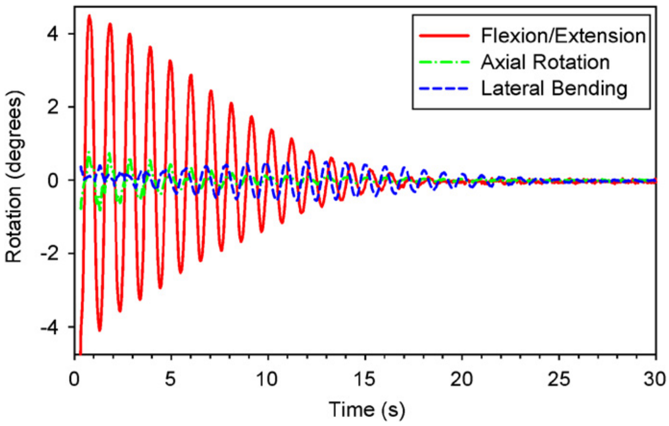

Fig. 2. Typical relative rotations for an FSU with a compressive pendulum load of 385 N and an initial rotation of 5° in extension.

Table 1
Average (±one S.D.) response of the FSUs ($n = 5$) for increasing axial compressive pendulum loads

Compressive load (N)	Period (s)	Natural frequency ($2\pi/s^{-1}$)	Bending stiffness (Nm/°)	Coefficient of damping (Nm/s)
78	0.61 (0.09)	10.4 (1.6)	1.7 (0.7)	1.4 (0.9)
181	0.81 (0.07)	7.9 (0.8)	2.3 (0.8)	1.9 (0.3)
282	0.92 (0.06)	6.8 (0.5)	2.5 (0.6)	2.3 (0.4)
385	0.97 (0.06)	6.5 (0.4)	3.0 (0.7)	3.8 (0.5)
488	1.00 (0.05)	6.3 (0.3)	3.5 (0.8)	4.0 (0.8)

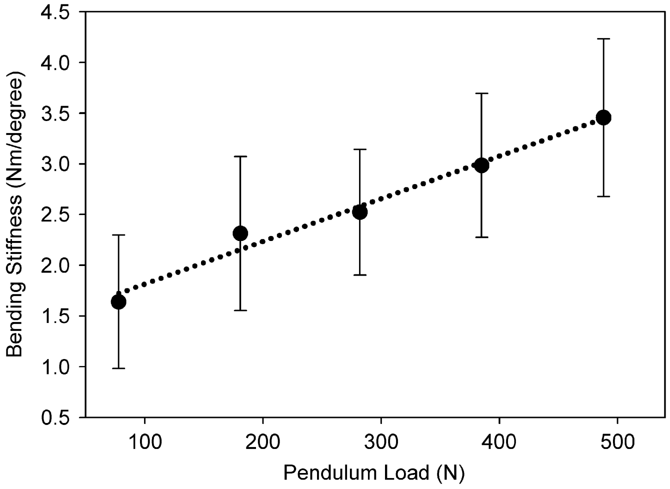

Fig. 3. Average (±one S.D.) FSU bending stiffness in flexion/extension increased significantly ($P < 0.01$) and correlated linearly ($R^2 = 0.99$) with increasing compressive pendulum load.

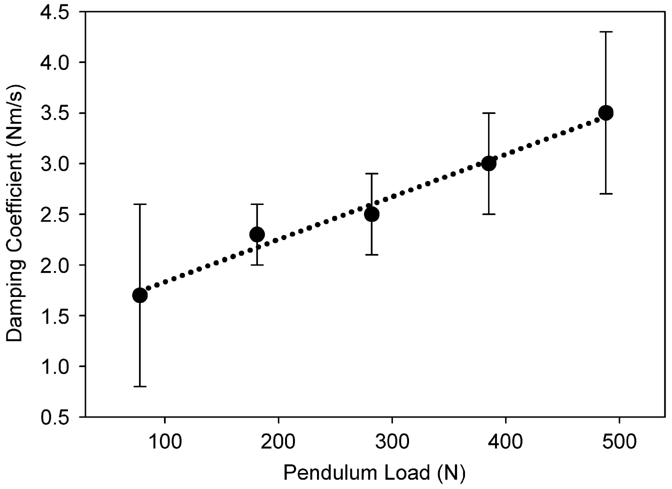

Fig. 4. Average (±one S.D.) coefficient of damping increased significantly ($P < 0.01$) and correlated linearly ($R^2 = 0.99$) with increasing compressive pendulum load.

largest load averaged 3.5 Nm/°. Likewise, the coefficients of damping correlated significantly with a linear ($R^2 = 0.98$, $P < 0.001$) with a linear increase in the compressive load (Fig. 4). The coefficient of damping in flexion tended to be greater than the coefficient of damping in extension (Fig. 5).

The experimental value for the bending stiffness and damping coefficients of the rotational bearing were in close agreement with the theoretical values of a frictionless pendulum system (Table 2). Theoretically (i.e. assuming an ideal frictionless bearing and no air resistance), the period is simply $2\pi\sqrt{I/mgl}$ and gives values of 1.2 and 1.4 s at the compressive loads of 78 and 488 N. The natural frequency is then 5.3 and 4.6 s^{-1} at these same compressive loads. The experimentally measured and computed values for the period of the pendulum with the rotational bearings were 1.3 and 1.4 s at these loads, with a natural frequency of 4.7 and 4.6 s^{-1}. Accordingly in an ideal rotational bearing the bending stiffness and damping coefficient are zero. We found values of bending stiffness of the rotational bearings to range from 0.08 to 0.007 Nm/° at the compressive loads of 78 and 488 N. For these same loads the damping coefficient was negligible but did increase from 0.02 to 0.04 Nm/s.

4. Discussion

A novel pendulum system for the study of the dynamic properties of human FSUs is described. Using simple physical pendulum theory and the observation that an FSU behaves viscoelastically (Hirsch, 1955), we modeled the FSU as an underdamped elastic vibrating system and studied its response to an initial perturbation of 5°. This approach allows the application of physiologic axial compressive loads during dynamic, unconstrained bending, which are not readily accomplished with many existing methods for determining the biomechanical properties of the ligamentous spine.

Verification of our approach comprised examining the response of the pendulum when it was supported by rotational bearings. In this configuration, we modeled the pendulum as an ideal pendulum with zero stiffness and damping. The experimental values we measured were in excellent agreement with this model. As the load increased, the period of the bearing supported pendulum more closely approached the theoretical values and the stiffness decreased. This is consistent with the observation that bearing performance is not ideal under light loads and improves with loading. The damping coefficient values

Table 2
Response of the pendulum when affixed to rotational bearings (values are the average of three trials)

Compressive load (N)	Period (s)		Natural frequency ($2\pi/s^{-1}$)		Bending stiffness (Nm/°)	Coefficient of damping (Nm/s)
	Exper.	Theoretical	Exper.	Theoretical		
78	1.3	1.2	4.8	5.3	0.08	0.02
181	1.3	1.2	4.7	4.8	0.07	0.02
282	1.4	1.3	4.6	4.7	0.05	0.03
385	1.4	1.4	4.6	4.6	0.02	0.04
488	1.4	1.4	4.6	4.6	0.01	0.04

Assuming and ideal frictionless rotational bearing, the theoretical values for period are simply $2\pi\sqrt{I/mgl}$, and are zero for bending stiffness and damping coefficient. The experimental parameters m, l and I are given in the Section 2.

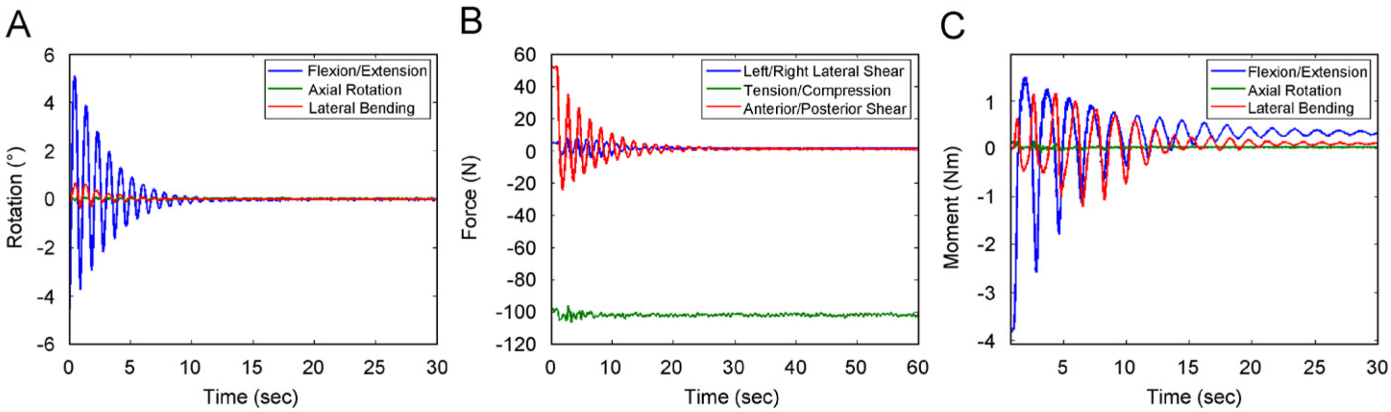

Fig. 5. For rotations (A) of an FSU with a compressive load of 181 N, the forces (B) and moments (C) recorded by the load cell oscillated but were not symmetrical. The translations movement of the FSU was negligible. The vertical axis of the compressive load channel of the load cell was zeroed with the weight of the pendulum frame, so the vertical loads in this graph are 78 N less than the actual value.

increased slightly with increasing compressive load. The close agreement between these experimentally measured values and those predicted supports the assumption of a dynamic physical pendulum and further that stiffness and damping values can be predicted.

Markolf and Steidel (1970) studied the dynamic properties of the thoracolumbar FSU in an approach somewhat similar to ours but with an important difference. They determined the free vibration response of the unloaded FSU by fixing the lower vertebral body, manually giving the upper vertebral body a rotary displacement of a few degrees, releasing it, and measuring the oscillation until it came to rest. In other words, they were "plucking" the upper vertebral body. They did not use a pendulum, and therefore were not able to apply a compressive load. The values for dynamic stiffness they determined ranged from 0.6 to 4.1 Nm/°. The lower range of values would be expected since they appear to have tested the spine within a smaller range of motion, conversely the upper values are surprisingly similar to ours considering this and the fact that compressive loads were not applied.

Interestingly, our dynamic bending stiffness values are similar in magnitude to those determined quasi-statically (Edwards et al., 1987; Gardner-Morse and Stokes, 2004; Miller et al., 1986; Schultz, 1979; White III and Panjabi, 1990). Stiffness coefficient values averaged across numerous studies are reported by White and Panjabi (1990) to be 1.4 and 2.0 Nm/° in flexion and extension, respectively, which are in close agreement with the values we report. Edwards et al. (1987) and Miller et al. (1986) reported stiffness values which were slightly larger than those presented in this study. This may be due to methodological differences: Miller et al. (1986) determined stiffness values using much larger extension rotations of approximately 9° and flexion rotations of approximately 12°. Edwards et al. (1987) applied axial compressive loads with nearly twice the magnitude of those in this study.

The linear correlation between bending stiffness and load reported in this study is consistent with previous studies (Edwards et al., 1987; Gardner-Morse and Stokes, 2004). Gardner-Morse and Stokes (2004) found a linear relationship between stiffness and axial preloading. In addition, Edwards et al. (1987) reported that FSUs loaded in flexion were stiffer at higher loads than at lower loads. Moreover, Janevic et al. (1991) found a linear decrease of flexibility (which can be interpreted loosely as the inverse of stiffness) with increasing preload.

The compressive preloads used in this study are in the lower end of the range considered to be physiologic. In an early *in vivo* study predicting the spine compression force for various activities of daily living, Nachemson et al. (1986) estimated values of 364 N for sitting arm out, 471 N for quiet standing, and 1243 N for standing while holding an 8 kg mass. The maximum compressive preload used in this study (488 N) is similar to that recommended for testing disc arthroplasty devices by ASTM (ASTM F2346) (500 N for lumbar devices). It is also similar to that used by some other researchers (Edwards et al., 1987; Gardner-Morse and Stokes, 2004), although less than that used by others (Janevic et al., 1991; Patwardhan et al., 1999).

Few studies report quantitative values of damping (Izambert et al., 2003; Markolf and Steidel, 1970), though quantification of this property provides insight into the mechanical nature of the spine (Hirsch, 1955; Izambert et al., 2003). Several studies suggest that the intervertebral disc has damping properties, and use analytical descriptions (Hirsch, 1955) or finite element models to characterize the damping characteristics (Goel et al., 1994; Kasra et al., 1992). With finite element models especially, several simplifying assumptions are made, but these validity of these assumptions remains untested. Our observation that the FSU behaves as an underdamped oscillating system is in general consistent with the work of Markolf and Steidel (1970). However, they calculated dimensionless damping factors without the influence of compressive loading that cannot be directly compared with values presented in this study. They also report a natural frequency of approximately 36 Hz. Without a compressive load, this value cannot be compared with our findings and also does not likely correlate with a physiological response.

Our study was limited because no attempt to quantify the degree of degeneration for the FSUs was made, although it has been shown that the level of disc degeneration can affect the biomechanical properties of the intervertebral disc (Haughton et al., 1999). Furthermore, we derived our equation of motion under the assumption that the intervertebral disc behaves as a linearly elastic and linearly underdamped system. Clearly the ligamentous spine behaves as a nonlinear elastic system: it has an initial value of low stiffness that increases with increasing motion until it reaches a relatively constant stiffness value (Panjabi et al., 1994). Since our computation of stiffness is based upon the dynamic response, our stiffness values are likely based on an average response that is an estimate of the final stiffness of the FSU, rather than an initial low stiffness.

We measured stiffness values of the FSU without measuring load values. This was accomplished by assuming the equation of motion of the system (Eq. (1)), and then calculating stiffness based upon the response to an initial perturbation. The data presented here is by no means demonstrative of the ability of such a system to accurately measure stiffness without a load cell, but it does present some initial data for such an approach in the dynamic study of ligamentous joints. Subsequent to this work we mounted the lower FSU potting to a six axis load cell and repeated these tests on a single specimen. The peak bending moment in flexion and extension reached averaged 6.2 ± 0.7 Nm across all five compressive loads. This would give a stiffness value of 1.2 Nm/°, in good agreement with our results and those of other researchers, but this remains to be further investigated.

The pendulum apparatus described addresses several challenges associated with *in vitro* biomechanical testing of the spine including dynamic application of bending moments, physiologic axial compressive loads, and unconstrained motion. To our knowledge, an apparatus capable of simultaneously performing these functions with the spine has not been reported previously. Furthermore, this approach is extremely cost-effective. Studying the spine under these conditions is crucial to understanding how it functions mechanically *in vivo*. This apparatus could also provide a means for performing dynamic, unconstrained testing of disc arthroplasty devices under physiologic loading conditions, which may allow designers of these devices to better replicate the dynamic response of the native disc.

Acknowledgments

This study was supported by the RIH Orthopaedic Foundation, Inc. and University Orthopedics, Inc.

References

Charnley, J., 1959. Lubrication of animal joints. Biomechanics 12–22.

Edwards, W.T., Hayes, W.C., Posner, I., White 3rd, A.A., Mann, R.W., 1987. Variation of lumbar spine stiffness with load. Journal of Biomechanical Engineering 109 (1), 35–42.

Gardner-Morse, M.G., Stokes, I.A., 2004. Structural behavior of human lumbar spinal motion segments. Journal of Biomechanics 37 (2), 205–212.

Goel, V.K., Goyal, S., Clark, C., Nishiyama, K., Nye, T., 1985. Kinematics of the whole lumbar spine. Effect of discectomy. Spine 10 (6), 543–554.

Goel, V.K., Voo, L.M., Weinstein, J.N., Liu, Y.K., Okuma, T., Njus, G.O., 1988. Response of the ligamentous lumbar spine to cyclic bending loads. Spine 13 (3), 294–300.

Goel, V.K., Park, H., Kong, W., 1994. Investigation of vibration characteristics of the ligamentous lumbar spine using the finite element approach. Journal of Biomechanical Engineering 116 (4), 377–383.

Goel, V.K., Wilder, D.G., Pope, M.H., Edwards, W.T., 1995. Biomechanical testing of the spine. Load-controlled versus displacement-controlled analysis. Spine 20 (21), 2354–2357.

Grassmann, S., Oxland, T.R., Gerich, U., Nolte, L.P., 1998. Constrained testing conditions affect the axial rotation response of lumbar functional spinal units. Spine 23 (10), 1155–1162.

Haughton, V.M., Lim, T.H., An, H., 1999. Intervertebral disk appearance correlated with stiffness of lumbar spinal motion segments. American Journal of Neuroradiology 20 (6), 1161–1165.

Hirsch, C., 1955. The reaction of intervertebral discs to compression forces. Journal of Bone and Joint Surgery (American) 37-A (6), 1188–1196.

Hirsch, C., Nachemson, A., 1954. New observations on the mechanical behavior of lumbar discs. Acta Orthopaedica Scandiavica 23 (4), 254–283.

Izambert, O., Mitton, D., Thourot, M., Lavaste, F., 2003. Dynamic stiffness and damping of human intervertebral disc using axial oscillatory displacement under a free mass system. European Spine Journal 12 (6), 562–566.

Janevic, J., Ashton-Miller, J.A., Schultz, A.B., 1991. Large compressive preloads decrease lumbar motion segment flexibility. Journal of Orthopaedic Research 9 (2), 228–236.

Jones, E.S., 1936. Joint lubrication. Lancet 1, 1043–1044.

Kasra, M., Shirazi-Adl, A., Drouin, G., 1992. Dynamics of human lumbar intervertebral joints. Experimental and finite-element investigations. Spine 17 (1), 93–102.

Markolf, K.L., Steidel, R.F., 1970. The Dynamic Characteristics of the Human Intervertebral Joint. In: Proceedings of ASME Annual Winter Meeting, New York.

Miller, J.A., Schultz, A.B., Warwick, D.N., Spencer, D.L., 1986. Mechanical properties of lumbar spine motion segments under large loads. Journal of Biomechanics 19 (1), 79–84.

Nachemson, A.L., Andersson, B.J., Schultz, A.B., 1986. Valsalva maneuver biomechanics. Effects on lumbar trunk loads of elevated intraabdominal pressures. Spine 11 (5), 476–479.

Panjabi, M.M., 1977. Experimental determination of spinal motion segment behavior. Orthopaedic Clinic North America 8 (1), 169–180.

Panjabi, M.M., 1988. Biomechanical evaluation of spinal fixation devices: I. A conceptual framework. Spine 13, 1129–1134.

Panjabi, M.M., Oxland, T.R., Yamamoto, I., Crisco, J.J., 1994. Mechanical behavior of the human lumbar and lumbosacral spine as shown by three-dimensional load-displacement curves. Journal of Bone and Joint Surgery (American) 76 (3), 413–424.

Panjabi, M.M., Kato, Y., Hoffman, H., Cholewicki, J., Krag, M., 2000. A study of stiffness protocol as exemplified by testing of a burst fracture model in sagittal plane. Spine 25 (21), 2748–2754.

Patwardhan, A.G., Havey, R.M., Meade, K.P., Lee, B., Dunlap, B., 1999. A follower load increases the load-carrying capacity of the lumbar spine in compression. Spine 24 (10), 1003–1009.

Schultz, A.B., 1979. Mechanical properties of human lumbar spine motion segments—Part I: responses in flexion, extension, lateral bending, and torsion. Journal of Biomechanical Engineering 101, 46–52.

Spenciner, D.B., Paiva, J.A., Crisco, J.J., 2003. Testing human cadaveric functional spinal units to the ASTM Draft Standard, "standard test methods for static and dynamic characterization of spinal artificial discs." Spinal implants: are we evaluating them appropriately, ASTM STP 1431, pp. 114–126.

Unsworth, A., Dowson, D., Wright, V., 1975. The frictional behavior of human Synovial joints—Part 1: natural joints. Journal of Lubrication Technology 369–376.

White III, A.A., Panjabi, M.M., 1990. Clinical Biomechanics of the Spine 722.

Wilke, H.J., Claes, L., Schmitt, H., Wolf, S., 1994. A universal spine tester for in vitro experiments with muscle force simulation. European Spine Journal 3 (2), 91–97.

Wilke, H.J., Rohlmann, A., Neller, S., Schultheiss, M., Bergmann, G., Graichen, F., Claes, L.E., 2001. Is it possible to simulate physiologic loading conditions by applying pure moments? A comparison of in vivo and in vitro load components in an internal fixator. Spine 26 (6), 636–642.
